# Comparative Evaluation of T-Cell Immune Response to BTV Infection in Sheep Vaccinated with Pentavalent BTV Vaccine When Compared to Un-Vaccinated Animals

**DOI:** 10.1155/2019/8762780

**Published:** 2019-12-02

**Authors:** Molalegne Bitew, Chintu Ravishankar, Soumendu Chakravarti, Gaurav Kumar Sharma, Sukdeb Nandi

**Affiliations:** ^1^Ethiopian Biotechnology Institute (EBTI), Ethiopia; ^2^Virus Laboratory, Center for Animal Disease Research and Diagnosis (CADRD), ICAR-Indian Veterinary Research Institute (IVRI), Izatnagar, Uttar Pradesh 243122, India; ^3^Division of Biological Products, ICAR-Indian Veterinary Research Institute (IVRI), Izatnagar, Uttar Pradesh-243122, India

## Abstract

Recent invasion of multiple bluetongue virus serotypes (BTV) in different regions of the world necessitates urgent development of efficient vaccine that is directed against multiple BTV serotypes. In this experimental study, cell mediated immune response and protective efficacy of binary ethylenimine (BEI) inactivated Montanide^™^ ISA 206 adjuvanted pentavalent (BTV-1, 2, 10, 16 and 23) vaccine was evaluated in sheep and direct challenge with homologous BTV serotypes in their respective group. Significant (*P* < 0.05) up-regulation of mRNA transcripts of IFN-*α*, IL-2, IL-6, IL-12, IFN-*γ* and TNF-*α* in PBMCs of vaccinated animals as compared to control (un-vaccinated) animals at certain time points was observed. On the other hand, there was a significant increase in mean ± SD percentage of CD8^+^ T cells after 7 days post challenge (DPC) but, the mean ± SD percentage of CD4^+^ T-cell population slightly declined at 7 DPC and enhanced after 14 DPC. Significant differences (*P* < 0.05) of CD8^+^ and CD4^+^T cells population was also observed between vaccinated and unvaccinated sheep. The vaccine also significantly (*P* < 0.05) reduced BTV RNA load in PBMCs of vaccinated animals than unvaccinated animals following challenge. There were no significant difference (*P* > 0.05) in cytokine induction, BTV RNA load and CD8^+^ and CD4^+^cell count among BTV-1, 2, 10, 16 and 23 serotype challenges except significant increase in mean ± SD percentage of CD8^+^ in BTV-2 group. These findings put forwarded that binary ethylenimine inactivated montanide adjuvanted pentavalent bluetongue vaccine has stimulated cell mediated immune response and most importantly reduced the severity of BTV-1, 2, 10, 16 and 23 infections following challenge in respective group.

## 1. Introduction

Bluetongue (BT) is an arthropod-transmitted hemorrhagic disease of wild and domestic ruminants. BT is list A disease and endemic in almost all the countries except Antarctica, concurrent with the geographic distribution, seasonal activity of competent Culicoides vector insects and appropriate climatic conditions [1, 2]. The disease is caused by Bluetongue virus (BTV) which is prototype species of the genus *Orbivirus* in the family *Reoviridae *[3, 4]. Currently, there are 27 recognized serotypes (BTV-1 to -27) with additions of the 25^th^ serotype (“Toggenburg orbivirus”) from Switzerland in goat and 26^th^ from Kuwait in sheep [2, 5–7] with recent identification of 27^th^ from Corsica, France in goat [8]. India is endemic with BT with first report in the year 1968 and now 23 out of 27 serotypes are prevailing in the country [9].

Due to wide antigenic heterogeneity among the serotypes, single strain of BTV in the vaccine does not offer cross protection. Like other arboviral diseases, BT is difficult to control using conventional bio-security measures. Hence, systematic vaccination is only effective tool to prevent clinical disease and virus spread. Conventionally, modified live virus (MLV) and inactivated vaccines have been used to limit the outbreaks of BTV in the world including in India.

Live attenuated vaccines have been used to control the disease spread in Europe till the year 2003 [10], however due to associated risks with live attenuated vaccines such as teratogenicity, reversion to virulence, immune-suppression and genetic reassortment, subsequently the use of inactivated vaccines were preferred [10–12]. Inactivated monovalent or polyvalent vaccines have been evaluated for its efficacy in control the spread of disease in India and other countries [10, 12–20].

Though inactivated vaccines confer protection largely through induction of neutralizing antibody, studies have shown that the suitable adjuvant(s) and booster dose can also induce cell mediated immune (CMI) response [21–23, 42]. Even there are reports of conferring protection by inactivated vaccines to BT without neutralizing antibodies mostly by through cytotoxic T lymphocytes (CTLs) [23]. Induction of both humoral and CMI response by inactivated vaccines would be highly beneficial in offering longer duration of protection to both homologous and heterologous serotypes [18, 23, 42].

Specific adjuvant such as Montanide^™^ ISA 206 VG has been reported to induce both B as well as T cell immune response. Hence it is advocated to assess the CMI response using suitable adjuvants in evaluating the vaccine efficacy. The level and potential role of CMI protection after vaccination with inactivated pentavalent BTV virus vaccine has not been estimated so far, hence the following study was taken up to comparatively evaluate the CMI response to the experimental BTV infection in vaccinated and un-vaccinated sheep.

## 2. Methods

### 2.1. Primer Design and Optimization of RT-qPCR Conditions

The sense and antisense gene-specific primer pairs for specific amplification of sheep cytokines IL-2, IL-6, IL-12, IFN-*α*, IFN-*γ*, TNF-*α* and housekeeping gene *β*-actin were designed by using Primer Quest Programme of Integrated DNA Technologies (IDT, Coralville, USA) (https://eu.idtdna.com/PrimerQuest/Home/Index) utilizing NCBI GenBank sequence information (http://www.ncbi.nlm.nih.gov/BLAST). All criteria required for SYBR/EVA Green qPCR were followed during primer designing. The primers were custom synthesized by M/s Eurofins Genomics Private Ltd., (Bangalore, India). All the primer sets were tested in gel based PCR to determine optimum annealing temperature for each primer set to be used in EVA/SYBER green based Real Time PCR. The details of primers, expected amplicon size and cyclic conditions are summarized in the Table 1.

### 2.2. Virus for Challenge and Vaccine Formulation

The pentavalent inactivated vaccine was produced as described earlier [18, 24]. In brief, BTV serotypes BTV-1 (TANUVAS isolate), BTV-2 (TANUVAS isolate), BTV-10 (Hyderabad isolate), BTV-16 (Hisar isolate) and BTV-23 (TANUVAS isolate) used in the production of pentavalent inactivated vaccine and for the challenge were received at the institute and revived in BHK-21 cell culture.

Virus isolates were harvested with appearance of complete cytopathic effects, generally between 4 and 5 days post infection. The serotypes were reconfirmed by RT–PCR targeting segment 2 using serotype specific primers as described earlier [2]. The virus in cell culture supernatant was concentrated by using 8% (w/v) PEG-6000 as per the standard procedure [24, 25]. The harvested virus was titrated following Reed and Munch method as described earlier [26] and then inactivated by 0.02 M BEI at 37°C for 48 h as described previously [24]. Once inactivation was completed, inactivated virus serotypes BTV 1, 2, 10, 16 and 23 were pooled in with equal TCID_50_. The pooled virus serotypes were tested for sterility and innocuity as per the standard procedures [24, 41], 208 ml of this pooled inactivated BTV was mixed with 242 ml of Montanide^™^ ISA 206 VG, (SEPPIC, France) adjuvant (water oil water emulsion on weight by weight basis) in 500 ml measuring cylinder and homogenized with homogenizer.

### 2.3. Animal Experimentation

The study was approved by the ethical committees of the Indian Veterinary Research Institute (IVRI). Power analysis was performed to estimate the number of animals in each group. A total of 27 apparently healthy native breed of sheep of 1.5–2 years of age obtained from IVRI farm, were confirmed for absence of antibodies against BTV by c-ELISA (Pourquier c-ELISA kit (IDEXX, UK)). Animals were dewormed and maintained in insect proof sheds. Animals were grouped randomly into three groups (i) vaccinated challenged (*n* = 15) (ii) unvaccinated challenged (*n* = 10) and (iii) unvaccinated unchallenged (*n* = 2) (Table 2).

The pentavalent vaccine formulation containing BTV serotypes 1, 2, 10, 16, and 23 prepared above was inoculated as 2 ml dose in each of the sub-group (*n* = 3) of animals of vaccinated infected group by subcutaneous (S/C) route at two sites neck and posterior thigh followed by booster dose on 21 days post vaccination (DPV). Animals of unvaccinated groups (*n* = 10) were inoculated with 2 ml of normal saline on 0DPV and 21DPV.

On 49 DPV, animals of vaccinated and unvaccinated groups were challenged by intradermal inoculation of 4 ml of clarified virus suspension of BTV-1, 2, 10, 16 and 23 serotypes passaged 3 times in BHK21 (clone 13) cells having titer of ≥10^6^ TCID_50_/ml to their respective groups of animals at multiple sites in the neck and under the thigh region. At the end of the experiment the animals were disposed off as per the recommended guidelines.

### 2.4. Rectal Temperature and Clinical Sign

Clinical symptoms and body temperatures were recorded and mean score were calculated as described previously [20]. The recordings were taken on 49DPV (0 DPC) (before inoculums injection), 50 DPV (1 DPC), 52DPV (3 DPC), 54DPV (7DPC), 57DPV (8DPC), 59DPV (10DPC), 62DPV (13DPC), 64DPV (15 DPC), 67DPV (18DPC) and 70DPV (21DPC).

### 2.5. Humoral Immune Response

Blood samples without anticoagulants were collected aseptically on 0, 3, 7, 10, 14, 21, 28, 38 52, 56, 63, 70, 120, 180 and 270 DPV through jugular vein puncture. The serum samples were separated and tested by the commercially available cELISA and the OD values were transformed to percentage inhibition (PI) values as described previously [20].

### 2.6. Quantification of Cytokine Transcripts by qRT PCR

Blood samples were collected using heparin (10 IU/ml) as anticoagulant on 0, 3, 7, 14 and 21 DPC. Mononuclear cell fractions were separated in the collected blood samples using Histopaque1077 (Sigma Aldrich) as per the standard procedures. Cells were suspended in 1 ml of RPMI and used for total RNA extraction. The PBMC pellet was re-suspended in 300 *μ*l of DEPC-PBS and transferred to a 2 ml DEPC treated microcentifuge tube. Total cellular RNA was extracted by Tri Reagent RT–4-bromoanisole method (Molecular Research Center, Inc., USA) following the recommended procedure. The RNA was dissolved in nuclease-free water. The purity of RNA was confirmed by optical density (OD) absorption ratio of OD 260 nm/OD 280 nm using Nanodrop spectrophotometer.

Total RNA (~1 *μ*g) of was reverse transcribed to cDNA with Oligo dT_18_ primers using RevertAid (reverse transcriptase) (200 units/*μ*l) (Fermentas, Lithuania) as per the recommendation of manufacturer. Reverse transcription was carried out at 42°C for 1 h followed by enzyme inactivation at 70°C for 10 min and cDNA was checked by gel based PCR using *β*-actin gene specific primers.

RT-qPCR was performed with Kappa SYBR^®^ Fast qPCR kit (Kappa Biosystems, Massachusetts, USA) using Mx3005P Real Time thermal cycler system (Stratagene, Agilent Technologies, USA) [27]. Each RT-qPCR reaction was put in duplicate in a total volume of 20 *µ*l, which contained 10 *µ*l of 2x Kappa SYBR^®^ Fast RT-qPCR master mix, 0.25 *µ*l each of 10 pmol forward and reverse primers, 1 *µ*l of cDNA template and rest nuclease free water. Forty cycles of denaturation at 95°C for 15 sec, annealing at different temperatures depending on the Tm of the primers for 30 sec and extension at 72°C for 30 sec were performed after an initial denaturation at 95°C for 15 min and last cycle at 95°C for 30 sec and at 65°C for 30 sec. Cycle threshold (Ct) values and amplification plot for all determined factors were acquired by using the “SYBR/EVA green (with dissociation curve)” method.

Relative expression of PCR product was quantified with the equation recommended by Pfaffl [28] using REST 2009 software (http://rest-2009.gene-quantification.info/). No template control reaction mix without any cDNA was kept to rule out reagents contamination. 0 DPC (49 DPV) values were used as calibrator for testing relative mRNA expressions of six cytokine transcripts in PBMCs taking *β*-actin as housekeeping gene.

### 2.7. Quantification of Viral Nucleic Acid

The presence of BTV RNA in blood samples were quantified at various intervals using RT-qPCR targeting BTV segment 5 (NS1) [2] which is highly conserved gene among BTV serotypes at 0 DPC (49 DPV) and 3, 7, 14 and 21 days post challenge. Total RNA was extracted from heparinized blood sample using Trizol-LS reagent (Life Technologies, USA) followed by precipitation and removal of cellular ssRNA by 2 M LiCl to obtain pure dsRNA [29, 30, 42]. 1 *µ*g of purified BTV RNA was converted into cDNA and used for standard curve preparation. Plasmid containing target gene was constructed to determine the initial concentration of cDNA and then to determine the PCR efficiency. A standard log_10_ dilution curve was undertaken using tenfold serial dilution of known copies of cDNA with corresponding *C*_t_ values. It was found that efficiency of the primer was 100%. Regression analysis of *C*_t_ values from each dilution resulted in regression coefficient (*R*^2^) of 0.99. All other RT-qPCR reaction procedures and thermal profiles were similar as described above except for the annealing temperature (Table 1).

### 2.8. Analysis of CD4^+^ and CD8^+^ T Cell Responses by Flow Cytometry

100 *µ*l of blood in EDTA was collected from immunized and unimmunized group of sheep at 0 days before challenge (49 DPV) and at 3DPC (52 DPV), 7 DPC (56 DPV), 14 DPC (63 DPV) and 21 DPC (70 DPV). 10 *µ*l of conjugated antibodies, mouse anti-ovine CD4: ALEXA FLUOR^®^647 (Neat-1/10) dilution) and mouse anti-ovine CD8: RPE (Serotec, Immunological Excellence, USA) were mixed and incubated at room temperature for 30 min. Cells were washed with PBS, lysed with RBC lysis buffer and fixed with conjugated antibodies. Fixed cell pellets were analyzed in FACS scan cytometer (Becton Dickinson, USA). Appropriate isotype controls, mouse IgG2a negative control: RPE (for CD8) and mouse IgG2a negative control: ALEXA FLUOR^®^647 (for CD4) (Serotec, Immunological Excellence, USA) were used to overcome background fluorescence, if any. Stained cells were acquired in FACS scan cytometer and analyzed using software CELLQuest version 3.1 (Becton Dickinson, USA) after subtraction of the corresponding isotype control. Ten thousand events were recorded from each sample. Mean percentage variation in peripheral blood was analyzed for lymphocyte subpopulation by RPE fluorescence at (FL-2) and ALEXA FLUOR^®^647 fluorescence at (FL-4).

### 2.9. Statistical Analysis

All the statistical analyses were performed using SPSS version 20 software program (SPSS Inc. Chicago, IL, USA). The statistically significant difference in the expression of cytokine transcripts, the difference between mean percentage of PBMC populations labeled by antibodies against CD4^+^ and CD8^+^, the differences between mean percentage of PBMC populations labeled by antibodies against CD4^+^ and CD8^+^ based on difference of the challenge serotype and the viral genome *C*_t_ value of the vaccinated and unvaccinated groups of sheep after challenge were analyzed using the nonparametric Scheirer–Ray–Hare technique for a two-way design with replication. *P*-value ≤ 0.05 was considered significant.

## 3. Results

The pentavalent vaccine formulation of BTV serotypes 1, 2, 10, 16, and 23 was assured to be free from bacteria, viruses, fungi or mycoplasma contamination by sterility test. The efficacy, especially the T-Cell response to the inactivated pentavalent vaccine formulation prepared in the recommended adjuvant was estimated by in-vivo experiments. Selected sheep were found sero-negative for BTV group specific VP7 antigen.

### 3.1. Animal Experimentation

None of the vaccinated sheep showed clinical signs after challenge during the observation period. Increased level of rectal temperature (mean = 40.8°C) and clinical sign were evident between 5 and 13 days post challenge (54–62 DPV) in control animals. There was significant difference (*P* < 0.05) between vaccinated and unvaccinated animals in the mean ± SD PI value, log10 mean ± SD of neutralizing antibody, mean rectal temperature and development of clinical signs after homologues virus challenge. However, there was no significant difference (*P* > 0.05) in all above parameters due to variability of challenge virus serotypes [20].

### 3.2. Humoral Immune Response

All sheep were sero-negative with mean ± SD percent inhibition (PI) value of 117.02 ± 25 in cELISA. After first vaccination with BEI inactivated montanide adjuvanted pentavalent BT vaccine, mean PI value was gradually declined in all vaccinated sheep indicating rise in the antibody titers. The pattern of sero-conversion was essentially similar to our previous study [20] with maximum titer was obtained on 28DPV.

### 3.3. Cytokine Transcripts Quantification

The efficiency of primers was determined by serial tenfold dilution of template cDNA in duplicate in real time PCR and it was found to be 99% for IFN-*γ*, 99.8% for IFN-*α*, 99.3% for IL-2, 100.2% for IL-6, 100% for IL-12, 101% for TNF-*α*, and 102.4% for *β*-actin.

**Figure 1:**
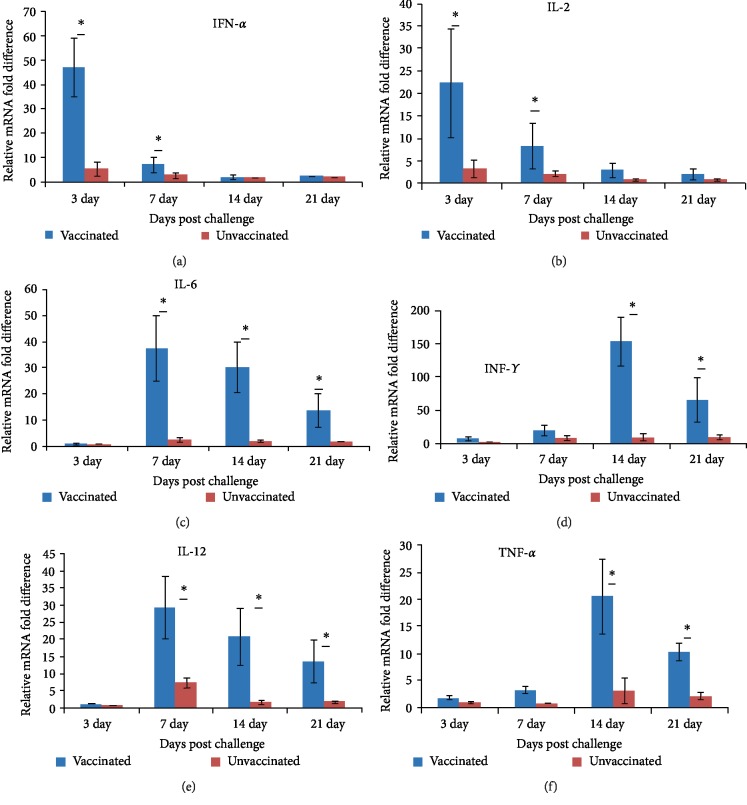
Graphs represent the level of expression of mRNA of six cytokine transcripts in vaccinated and unvaccinated sheep following challenge. The difference in the expression level was calculated by Pfaffl method using REST 2009 software after test samples were standardized with endogenous housekeeping *β*-actin gene and calibrator (uninfected controls). Vaccinated sheep (*n* = 15) were expressed all six cytokines significantly high (*P* < 0.05) compared to unvaccinated sheep (*n* = 10) following challenge of BTV-1, 2, 10, 16 and 23 to their respective group. The data was analyzed with Scheirer-Ray-Hare technique. ∗ indicates *P* < 0.05.

In this study it was found that both vaccinated and unvaccinated sheep exhibited enhanced expression of IFN-*α*, IL-2, IL-6, IL-12, IFN-*γ* and TNF-*α* mRNA transcript following challenge with individual BTV serotypes. However, compared to unvaccinated sheep, vaccinated sheep showed significant (*P* < 0.05) up-regulation of these cytokines (Figures 1(a)–1(f)).

IFN-*α* and IL-2 showed significant (*P* < 0.05) up regulation at 3DPC, which gradually declined at days 14 and 21, post challenge (Figures 1(a) and 1(b)). On the other hand, there was significant up-regulation in expression of IL-6 and IL-12 which peaked at 7 DPC and gradually declined at 14 and 21 DPC in vaccinated animals compared to unvaccinated sheep. However, the most profound up regulation of IFN-*γ* and TNF-*α* transcript expression were at 14 DPC in vaccinated animals which gradually declined at 21 days in comparison to Figures 1(c)–1(f)). Unlike unvaccinated sheep, vaccinated sheep clearly demonstrated significant (*P* < 0.05) up-regulation of IFN-*γ*, IL-6, IL-12 and TNF-*α* in PBMC at 14 DPC and in case of IFN-*α*, IL-2 at 3 DPC. There was no significant differences (*P* > 0.05) in cytokine induction between BTV-1, 2, 10, 16 and 23 serotype challenges (data not shown).

### 3.4. Viral Nucleic Acid Quantification

BTV RNA load was monitored in heparinized blood of both vaccinated and unvaccinated groups to measure the efficacy of the vaccine in reducing the viral loads in the blood of sheep following challenge. On the contrary to vaccinated sheep, in all unvaccinated sheep viral genome copies were detected using real-time RT-PCR assays at least once or more times between from 3 to 21 DPC (Table 3). On 7^th^ DPC, 4 vaccinated sheep challenged by BTV-1, 2, 10 and 23 showed viral genome copies with higher *C*_t_ values of 38, 36, 34 and 35 respectively compared to un-vaccinated sheep suggesting the transient viraemia of low titer (Table 3).

### 3.5. Analysis of CD4^+^ and CD8^+^ T Lymphocyte

**Figure 2:**
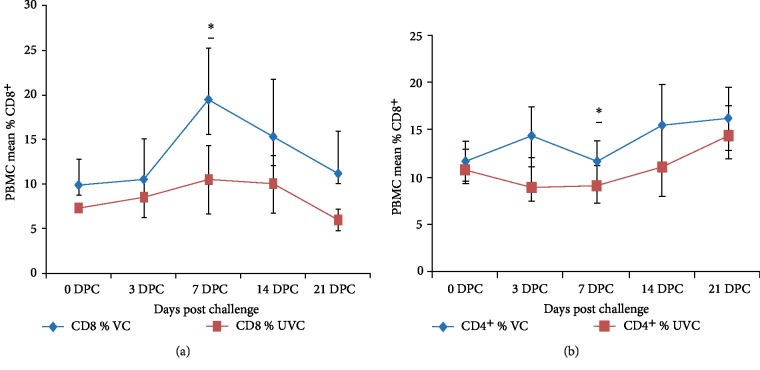
Mean ± SD percentage of PBMC populations labeled by antibodies against CD8^+^ (a) and CD4^+^ (b) T-lymphocytes of vaccinated and unvaccinated animals after challenge throughout the experiment. There was a significant difference between vaccinated and unvaccinated animals by Scheirer–Ray–Hare technique in their PBMC population labeled by antibodies against CD4^+^ and CD8^+^. Abbreviations: VC = Vaccinated challenged (*n* = 15), UVC = Unvaccinated challenged (*n* = 10), DPC = Days post challenge. ∗ indicates *P* < 0.05.

To evaluate the protective efficacy of cellular immune response, the proportion of CD4^+^ and CD8^+^ T lymphocytes numbers in PBMC of vaccinated and unvaccinated sheep at different time intervals were tested (Figures 2(a) and 2(b)).

The mean ± SD percentage of CD8^+^ T lymphocytes at 0 DPC (49 DPV) was 9.83 ± 3 and 7.23 ± 1.3 for vaccinated and unvaccinated sheep respectively (Figure 2(a)). It was gradually enhanced from 3 days post challenge with a peak at 7 DPC and gradually declined at 21 DPC. There was a significant difference (*P* < 0.05) between vaccinated and unvaccinated animals in the mean ± SD percentage of CD8^+^ T cells in PBMC fraction which was very high at 7 and 14 DPC (Figure 2(a)).

Mean ± SD percentage of CD4^+^ T lymphocytes was 11.68 ± 2.2 and 10.85 ± 1.2 of PBMC at 0 DPC for vaccinated and unvaccinated animals respectively (Figure 2(b)). After 3 DPC, the level declined and reaching 10.57 ± 2.2 and 9.08 ± 1.8 on day 7 for vaccinated and unvaccinated animals respectively. The level then showed increment for the rest of the study. There was a significant difference (*P* < 0.05) between vaccinated and unvaccinated animals in their CD4^+^ T lymphocytes proportion in PBMC fraction (Figure 2(b)).

**Figure 3:**
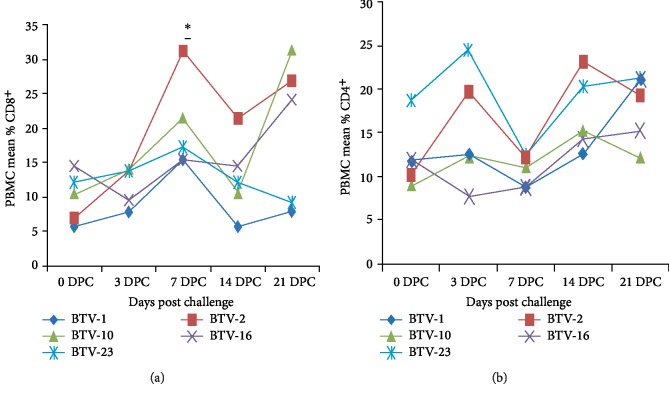
Mean percentage of PBMC populations labeled by antibodies against CD8^+^ (a) and CD4^+^ (b) of vaccinated sheep challenged (*n* = 3 in each group) with different viruses throughout the experiment. There was no significant difference (*P* > 0.05) between PBMC population labeled by antibodies against CD4^+^ and CD8^+^ with the difference in the challenge serotype except significantly increased mean ± SD PBMC populations labeled by antibodies against CD8^+^ were registered by BTV-2 challenge based on Scheirer-Ray-Hare technique. ∗ indicates*P* < 0.05.

The Mean percentage of PBMC population labeled by antibodies against CD8^+^ and CD4^+^ of vaccinated and challenged sheep with different serotype viruses were tested by nonparametric Scheirer–Ray–Hare technique and found that there was no significant difference (*P* > 0.05) in the mean percentage of PBMC labeled by antibodies against CD8^+^ and CD4^+^ between different BTV serotype challenges (BTV-1, 2, 10, 16 and 23) except BTV2 showing significantly (*P* < 0.05) high mean percentage of PBMC labeled by antibodies against CD8^+^(Figures 3(a) and 3(b)).

## 4. Discussion

Bluetongue is an infectious, noncontagious arthropod bore viral disease of wild and domestic ruminants. It is transmitted by culicoides midges and is prevalent in sub-tropical, tropical and temperate climates between latitude 53°N and 35°S (ref to be cited). There are 27 serotypes of BTV (ref to be cited). The multiplicity of serotypes possessing poor to moderate antigenic and cross-protective potential has caused the task of protecting the sheep against BT, rather intricate. There is an urgent need to determine serotype, as serotypes under a particular nucleotype would confer protection against not only homologous serotypes but also against heterologous serotypes [18, 19]. BEI-inactivated Montanide adjuvanted pentavalent vaccine has been shown to confer protective immune response against challenges with homologues BTV serotypes 1, 2, 10, 16 and 23 (ref to be cited). Montanide adjuvant was also able to enhance vaccine efficiency in terms of titer and period of immune response. It has been exhibited that it is adjuvant of choice to encourage both humoral as well as cellular responses (ref to be cited).

In this study both vaccinated and unvaccinated sheep exhibited significant expression of IFN-*α*, IL-2, IL-6, IL-12, IFN-*γ* and TNF-*α* transcripts following challenge. However, PBMC of vaccinated sheep showed significant up-regulation of these cytokines compared to unvaccinated ones indicating the role of these cytokines in minimizing the viraemia and preventing the clinical sign development. This result is in line with findings of different authors. Umeshappa et al. [18] reported the expression of IL-2, IL-12, IFN-*α* and IFN-*γ* in Indian native sheep after vaccination with binaryethylenimine (BEI)-inactivated BTV-1monovalent vaccine and challenged with heterologous BTV-23serotype. Ruscanu et al. [31] (2012) also reported the significant expression of IFN-*α*/*β* and other proinflammatory cytokines including TNF-*α*, IL-6, and IL-12p40 with challenge of BTV2 and BTV8 at 6 DPC. There were no significant differences in IFN-*α*/*β* induction between two BTV serotypes (*P* = 0.6). Channappanavar et al. [32] reported low induction of IL-12, IFN-*γ* and TNF-*α* cytokine mRNA in lymph nodes (DLN), spleen and PBMCs during the 8 DPI and gradually increased around 15 DPI.

In this study, IFN-*α* and IL-2 were significantly up regulated at 3 DPC and gradually declined at days 14 and 21 post challenge. This is because, IL-2 and IFN-*α* expression were associated with the CD4 and CD8 T cell responses both in vaccinated and unvaccinated sheep. IFN-*α* is secreted by leukocytes and other cells having antiviral and fever induction ability whereas, IL-2 secreted by Th1 lymphocytes and is important for proliferation and differentiation of T lymphocytes. Therefore, vaccination has induced elevated secretion of these cytokines in PBMC of vaccinated animals compared to nonvaccinated animals following challenge [18]. In contrast, IL-12, IL-6, IFN-*γ* and TNF-*α* were expressed in PBMC of both vaccinated and unvaccinated animals after 7 DPC. Unlike unvaccinated sheep, immunized sheep demonstrated significant up-regulation of IFN-*γ*, IL-6, IL-12 and TNF-*α* in PBMC. IL-6 is secreted by Th2 cells, macrophages and dendritic cells targets proliferating B cells and plasma cells to promote differentiation to plasma cells. IL-12 are a pro-inflammatory cytokine that play a crucial role in the induction of adaptive CD4^+^ and CD8^+^ T cell responses by enhancing IFN-*γ* production [33]. As in other viral infections, IL-12 is also produced by BTV-infected dendritic cells [34]. IFN-*γ* is a pleiotrophic pro-inflammatory cytokine produced mainly by NK cells, cytotoxic T cells (CTLs) and also Th1 cells in response to an antigenic stimulus [35]. IFN-*γ* is known to play an important role in regulating the adaptive immune response against viral antigens [32, 36]. Initial reduction in IFN-*γ* levels could be due to loss of CD4^+^ and CD8^+^ T cells associated with high levels of IFN-α in initial stage of BTV infection [18, 32]. But the possibility of an early increase in IFN-*α* sensitizing CD8^+^ T cells to produce IFN-*γ* during later stages of infection cannot be ruled out [32, 37, 38].TNF-*α* is known to be secreted by macrophages, Thl cells, mast cells and antigen-specific CTLs when encountering viral antigens which help in proliferation and differentiation of T cells, B cells, macrophages, NK cells and fibroblasts [32]. Presently, TNF-*α* activity in the infected animals positively correlated well with the CD8^+^ T cell frequency. Perhaps CD8^+^ T cells might have undertaken TNF-*α* production during later stages of infection.

In the present investigation, 4 of vaccinated sheep challenged with BTV-1, 2, 10 and 23 showed viral genome copies with high *C*_t_ value on 7 DPC compared to unvaccinated sheep, suggesting transient viraemia of low titer and there were no evident clinical signs. Unvaccinated animals were able to display detectable BTV-RNA genome amplification by BTV specific qPCR starting from 3DPC. There was significant difference (*P* < 0.05) between vaccinated and unvaccinated sheep in the level of BTV-RNA genome detected by qPCR assay. Monitoring the level of viraemia in vaccinated animals after challenge infection is considered the most effective way to evaluate vaccine efficacy [12, 14, 18, 19]. However, in the light of the low amount of viral genome detected only at 7^th^ DPC and the absence of clinical disease, the potential epidemiological relevance of this finding is highly important. At this low level of circulating viral genome, the presence of infectious virus would not be demonstrated and insect vector would be highly unlikely to become infected [14, 19, 39]. These findings suggest that this vaccine may prevent both virus dissemination and disease spread from vaccinated to susceptible animals. Therefore, in the presence of vector-borne BTV serotype 1, 2, 10, 16 and 23 the inactivated vaccine not only prevents virus replication but also effectively induces a protective immune response. The finding of this study is in line with the works of Eschbaumer et al. [14] who observed one vaccinated animal, having BTV genome with a C_t _of 36 in the highly sensitive BTV-8-specific real-time RT-PCR on day 10, but the animal was negative in subsequent samplings. Umeshappa et al. [19] also reported the viral load of 1.32 log_10_ in vaccinated sheep at 7 DPC which was lower than unvaccinated sheep. On contrary, Umeshappa et al. [19] detected no viral load in vaccinated sheep at any other point of time and this probably explains the transient viraemia and significant reduction in clinical and pathological scores observed in vaccinated sheep compared to unvaccinated ones. On the contrary, de Diego et al., [40] detected no BTV-RNA genome by BTV-specific qPCR in blood after challenging vaccinated animals with BTV serotype 1 [40].

CTL, an important component of CMI, is indispensable for protection against viruses and is often intermediary of cross protective immunity [21]. Anti-BTV CTL showing serotype cross-reactivity have been detected in mice and sheep with peak CTL response observed between 7 and 21 DPC [19, 40]. In the present study, enhanced activity of CMI was observed at 7 DPC which in lined with high Ct value of viral load in vaccinated sheep. CD8^+^ T cell response was also significantly different between vaccinated and unvaccinated animals. However, the viral load detected in vaccinated sheep was lower than the unvaccinated one's and this could be due to the enhanced activity of CD8 T-lymphocytes in killing the infected cells in vaccinated sheep. Further, it probably explains the transient viraemia and significant reduction in average clinical score (ACS) values observed in vaccinated sheep than unvaccinated ones (data not shown).

In the present study the mean ± SD percentage of CD4^+^ T lymphocytes represented 11.68 ± 2.2 and 10.85 ± 1.2 of PBMC at 0 DPC for vaccinated and unvaccinated animals respectively. After challenge, the level decreased significantly between days 3 and 7 and then enhanced for the rest of the trial. There was a significant difference (*P* < 0.05) between vaccinated and unvaccinated animals in the CD4^+^ T proportion. This result is in agreement with de Diego et al. [40] and Umeshappa et al. [19] who found low level of CD4^+^ T cells after challenge. All the above data put forward the evidence of development of enhanced CMI in BEI-inactivated montanide adjuvanted pentavalent bluetongue vaccination in sheep.

## Figures and Tables

**Table 1:** Oligonucleotide primer sequences and optimal amplification conditions of RT-qPCR reaction for cytokines, *β*-actin and BTV.

Set	Primer name	Primer sequence (5′-3′)^a^	Product size (base)	Primer length (base)	Anneling *T*° (°C)	Primer binding position	Accession number
1	IFN-*α* FP	ACCTTCCAGCTCTTCAGCACAGA	187	23	60.0	190–212	AY802984
	IFN-*α* RP	TGTGGAAGTGTTTCCTCACAGCCA		24	60.4	353–376	
2	IFN-*γ* FP	CTTGAACGGCAGCTCTGAGAAACT	91	24	59.0	367–390	X52640
	IFN-*γ* RP	ATTGATGGCTTTGCGCTGGATCTG		24	60.0	434–457	
3	IL-2 FP	CAAACTTCTAGAGGAAGTGCTAGAT	76	25	59.7	192–216	X60148
	IL-2 RP	GTCCATTGAATCCTTGATCTCTCT		24	57.2	244-267	
4	IL-6 FP	ACTGCTGGTCTTCTGGAGTA	100	20	57.3	364–383	X68723
	IL-6 RP	TTCTGATACTGCTCTGCAACTC		22	58.4	442–463	
5	IL-12 FP	TCTTCACAGACCAAACCTCAGCCA	111	24	60.0	863–886	NM001009438
	IL-12 RP	ACACAGATGCCCATTCACTCCAGA		24	60.0	950–973	
6	TNF *α* FP	TGGGCCAACTCCCTCTGTTTATGT	163	24	59.5	2195–2218	EF446377
	TNF *α* RP	AGTTTGTGTCTCCCAGGACACCTT		24	59.9	2291–2314	
7	*β*-actin FP	TGAAGATCCTCACGGAACGTGGTT	84	24	59.9	305–328	AF129289
	*β*-actin RP	AGCAGAGCTTCTCCTTGATGTCAC		24	58.5	365–388	
8	Seg5 (NS1) FP	GCAGCATTTTGAGAGAGCGA	101	20	59	169–188	JQ740775
	Seg5 (NS1) RP	CCCGATCATACATTGCTTCCT		21	58	249–269	

^a^Primers were designed based on sequences of ovine cytokine genes from GenBank database.

**Table 2:** Details of sheep involved in the cytokine expression and CMI study.

Vaccine group	Vaccinated/challenged	Unvaccinated/challenged	Unvacinated/unchallenged	Challenge virus
1	3	2	2	BTV-1
2	3	2		BTV-2
3	3	2		BTV-10
4	3	2		BTV-16
5	3	2		BTV-23

Total	15	10	2	27

**Table 3:** Threshold cycle (*C*_t_) values of BTV specific qPCR results for vaccinated and unvaccinated sheep.

Serial no	Sheep code	Vaccine group^∗^	Days post challenge (DPC)					BTV challenge serotype
			0	3	7	14	21	
1	4	VC	No *C*_t_	No *C*_t_	38.17	No *C*_t_	No *C*_t_	1
2	14	VC	No *C*_t_	No *C*_t_	36.5	No *C*_t_	No *C*_t_	2
3	24	VC	No *C*_t_	No *C*_t_	No *C*_t_	No *C*_t_	No *C*_t_	10
4	34	VC	No *C*_t_	No *C*_t_	No *C*_t_	No *C*_t_	No *C*_t_	16
5	44	VC	No *C*_t_	No *C*_t_	No *C*_t_	No *C*_t_	No *C*_t_	23
6	3	VC	No *C*_t_	No *C*_t_	No *C*_t_	No *C*_t_	No *C*_t_	1
7	13	VC	No *C*_t_	No *C*_t_	No *C*_t_	No *C*_t_	No *C*_t_	2
8	23	VC	No *C*_t_	No *C*_t_	No *C*_t_	No *C*_t_	No *C*_t_	10
9	33	VC	No *C*_t_	No *C*_t_	No *C*_t_	No *C*_t_	No *C*_t_	16
10	43	VC	No *C*_t_	No *C*_t_	No *C*_t_	No *C*_t_	No *C*_t_	23
11	5	VC	No *C*_t_	No *C*_t_	No *C*_t_	No *C*_t_	No *C*_t_	1
12	15	VC	No *C*_t_	No *C*_t_	No *C*_t_	No *C*_t_	No *C*_t_	2
13	51	VC	No *C*_t_	No *C*_t_	No *C*_t_	No *C*_t_	No *C*_t_	10
14	35	VC	No *C*_t_	No *C*_t_	No *C*_t_	No *C*_t_	No *C*_t_	16
15	45	VC	No *C*_t_	No *C*_t_	No *C*_t_	No *C*_t_	No *C*_t_	23
16	9	UVC	No *C*_t_	No *C*_t_	22.94	No *C*_t_	No *C*_t_	1
17	17	UVC	No *C*_t_	No *C*_t_	24.05	26.5	No *C*_t_	2
18	29	UVC	No *C*_t_	22.3	28.27	35.42	26.5	10
19	37	UVC	No *C*_t_	No *C*_t_	24.55	26.55	28.01	16
20	48	UVC	No *C*_t_	No *C*_t_	25.63	No *C*_t_	No *C*_t_	23

^∗^There was significant difference (*P* < 0.05) between vaccinated and unvaccinated animals in their BTV RNA detection analyzed by Scheirer-Ray-Hare technique. Abbreviations: VC = Vaccinated challenged (*n* = 15), UVC = Unvaccinated challenged (*n* = 5).

## Data Availability

The data is available and can be provided on demand.
